# Exploring informal science education responses to COVID-19 global pandemic: learning from the case of the Gwacheon National Science Museum in Korea

**DOI:** 10.1007/s11422-021-10073-5

**Published:** 2022-03-18

**Authors:** Myeong Ji Kim, Da Yeon Kang, Sonya N. Martin

**Affiliations:** Seoul National University, Seoul, Republic of Korea

**Keywords:** Informal education, Science museums, Pandemic, Online content, Marginalized learners, 비형식 교육, 과학관, 판데믹, 온라인 콘텐츠, 소외된 학습자

## Abstract

Science museums have long been heralded as important informal science education sites where people can engage in voluntary and experiential science learning. In this paper, we identify and raise questions about how science museum responses to a global pandemic could impact on accessibility of informal science education for the public. To explore these issues, we examined the response of the Gwacheon National Science Museum (GNSM) to COVID-19 in South Korea using publicly available data from the museum website and museum YouTube video channel. Analysis shows that the pandemic has increased and diversified the GNSM’s provision of science content for the general public via online platforms, such as YouTube and the museum website. In addition, GNSM educators are preparing special outreach education projects for deaf and blind visitors, who have often been excluded from informal science learning opportunities. By discussing these changes, we seek to raise questions about the potential for a global pandemic, like COVID-19, to affect informal science learning opportunities for a diverse group of people.

## Korean abstract

과학관은 비형식 과학교육 기관으로, 다양한 사람들이 자발적이고 체험적인 과학 학습을 할 수 있는 공간으로서의 역할을 수행해왔다. 본 논문에서는 COVID-19로 인한 판데믹 상황에서 과학관이 교육 기능을 지속하기 위해 어떻게 대응했는지 살펴보았다. 이를 위해 한국의 국립 과학관 중 하나인 국립과천과학관을 대상으로 과학관 웹 사이트와 과학관 유튜브 채널에 공개된 데이터를 사용하여 분석을 진행했다. 또한, 기존 국립과천과학관의 프로그램 중 소외된 학습자였던 청각 및 시각장애인을 위하여 운영했었던 교육 프로그램을 조사했다. 본 논문은 과학관이 판데믹 상황에서 수행한 과학교육 활동에서의 변화를 기술하고, 이로써 향후 다양한 방문객 집단의 학습에 어떤 영향을 미칠지 탐색하고자 한다.

Science museums offer important opportunities for visitors to develop a positive attitude toward science and can improve scientific literacy in terms of public understanding of science and public participation in science (Schwan et al., 2014). Science museums play a major role as centers for communication between the public and science, and as places for satisfying people’s curiosity. Many science education studies have focused on science museums’ roles for school-age students and children, but in recent years science museums have become a place for people of all ages that can support various types of group learning. Activities in science museums are mainly voluntary, so visitors can control their learning according to their interests (Anderson et al., 1998). According to John Falk and Lynn Dierking (2007), when learning takes place in a free-choice situation such as a museum, learners show more intrinsic synchronization and a more solid sense of self-respect, attribution, and internal and external control of their learning.

In the last two decades, increasingly science museums have been seen as places that can serve lifelong learning in terms of free-choice learning for visitors of all ages. The greater the autonomy the visitors have in learning, the more successful the results can be. In addition, many activities in science museums include experiential learning, which has been shown to heighten students’ curiosity and enjoyment of science while also helping to expand student knowledge and skills and provide new perspectives on science (Falk et al., 2004). Informal science education has been seen an important venue for realizing science learning opportunities “for all”. This is because informal science education can embrace a greater variety of learners in a less restricted educational environment than is possible in formal school settings, which require particular formats, systems, and regulations (Gi et al., 2019).

However, since December 2019, when a novel coronavirus (COVID-19) emerged, science museums all over the world have been facing challenges. On March 11th, 2020, after more than 118,000 cases had been confirmed in 114 countries, the World Health Organization [WHO] declared a global pandemic (WHO, 2020). This announcement resulted in the closure of museums all over the world. The shutting down of museums has had a huge impact on the economic stability of museums as they require funds to support educational programs and for on-going maintenance of facilities and exhibits. To assess the impact of COVID-19 on museums, the International Council of Museums [ICOM] (2020) surveyed workers in 1,600 science museums in 107 countries from April 7 to May 7, 2020 to ask about the impact the lockdown has had on workers and museums.

Survey results showed that nearly all (94.7%) of the 1,600 science museums were closed, and the vast majority of museums (81.5%) reported that less than one-quarter of their staff members were still working. The ICOM reported a long-lasting negative outlook for museums in terms of economic impact as only about one-third of respondents (36.8%) reported that staff employment was not expected to decrease as a result of the closures. Further, the majority of respondents (82.6%) reported that educational programs would decrease and a little less than half of the respondents (40.4%) expected a decrease in public funding. The report also found that more than half (54.4%) of the museums anticipated short-term workers, including freelancers and contract workers, would be negatively affected if museum closures continued. By late summer 2020, a study reported more than one-third of all museums in the United States there was a significant risk of permanent closure within the next year (American Alliance of Museums, 2020).

As of mid-July 2021, the COVID-19 virus has rapidly spread with over 188 million confirmed cases and a worldwide death toll of over 4.0 million people (WHO, 2021). For now, people are largely trying to adjust and adapt to living in this pandemic situation, and many have accepted it as the “new normal”. In the meantime, educators everywhere are struggling to find alternative ways to teach. This includes educators working at science museums. Unlike formal education systems that have been able to transition to online education or blended education methods to support students’ learning, science museums are primarily designed to facilitate visitor learning through interactions with on-site exhibitions and education programs. Thus, it will not be simple for educators to respond to the closure of museums as the educational programming has been purposefully designed to have visitors interact with and experience artifacts and exhibits in the museum setting. However, museum educators all around the world are rapidly working to shift their educational offerings and services in response to the pandemic (ICOM, 2020).

We believe that this incredibly difficult situation has the potential to significantly re-shape how informal educators working in science museums will meet the learning needs of visitors in the future, whether they are visiting in real life or virtually. Similar to science museums internationally, science museums in Korea have been forced to rapidly respond to forced closure during the pandemic. In this paper, we first describe the general impact of COVID-19 on science museums in Korea. Then we present a more in-depth description of response measures taken by educators at Gwacheon National Science Museum (GNSM) as an example of how educational responses to the pandemic have the potential to shift how visitors access science museum content and how visitors engage in informal learning experiences. We conclude this paper by raising questions about the ways in which the pandemic may serve to re-frame current thinking about informal science teaching and learning and the roles and responsibilities of science museums.

## General response to the pandemic by science museums in Korea

In Korea, science museums are run as professional informal science education institutes by staff members that collect, preserve and display scientific equipment and technological data and who offer various education programs to disseminate scientific and technological knowledge (for more information, see the *Establishment, Operation, and Promotion of Science Museum Act*, passed by the Korean Ministry of Science and Information, Communication, Technology in 2013). Before the pandemic, science museums primarily provided on-site exhibitions and various science education programs for professionals and visitors of all ages. Following the WHO’s declaration of the global pandemic in March 2020, Korea initially experienced a rapid increase in the number of people who tested positive for the virus. Due to previous experiences with contagious viruses, such as MERS and H1N1, the Korean government had already developed a national health alert system to reduce the spread of infectious diseases.

On February 23, 2020, the government raised the crisis alert to the highest level, which required that public offices and facilities be closed. As national science museums are funded by the government and are considered to be a public office, they are subject to legislation requiring their closure during a national health crisis. During the first half of 2020, the alert system remained at the highest level (red), but over the last year, the government has implemented different mandates for regulating public spaces in different areas of the country—dependent upon the severity of the outbreak. According to a survey by the Korean Science Center and Museum Association [KSCMA] (2020), by mid-March, 115 out of a total of 137 science museums had closed to the public. The following week, the government released statements about the importance of intensifying restrictions and urged citizens to refrain from attending meetings, eating out at restaurants, or traveling unnecessarily. During this time, the government asked that religious facilities, indoor sports facilities, and entertainment facilities all remain closed. After four weeks, the government eased the restrictions starting from April 20 to allow outdoor public facilities, such as national parks and arboretums, to re-open. However, science museums were to remain closed until May 6. After this time, public facilities, including science museums were allowed to partially re-open if they implemented measures to limit the number of visitors in compliance with government guidelines.

**Fig. 1:**
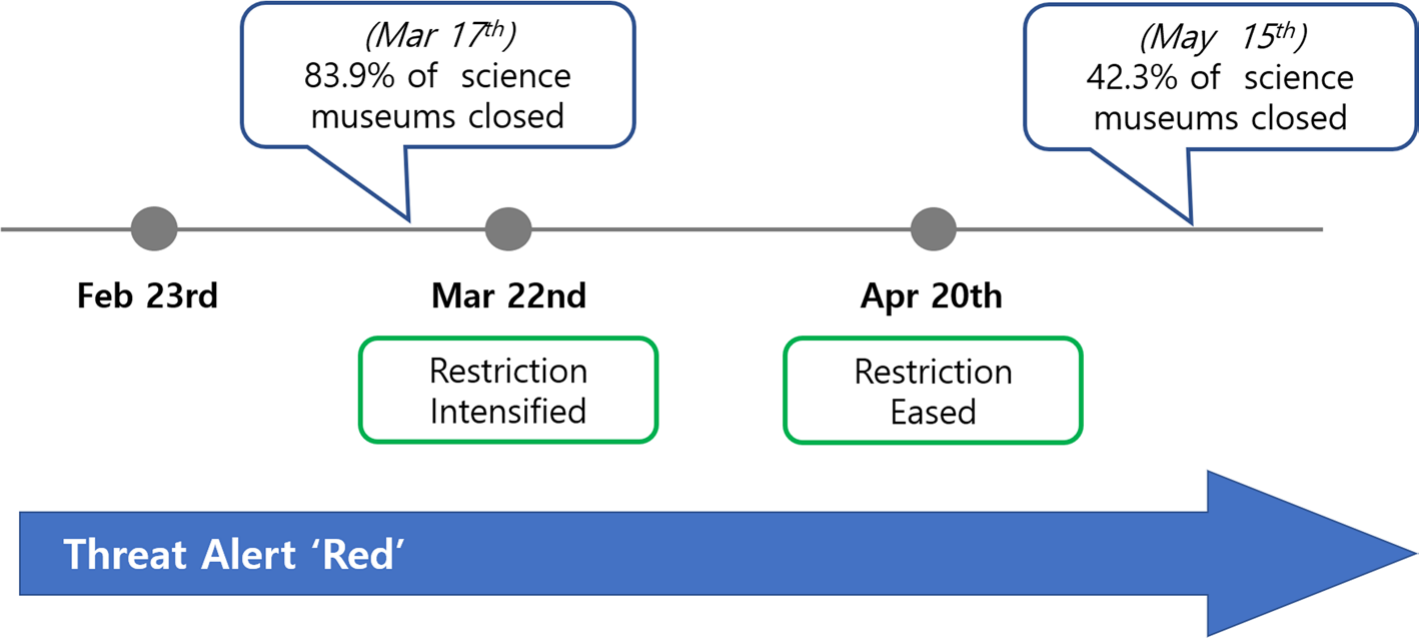
Science museums in Korea were repeatedly opened/closed in Spring 2020

For example, museums were required to open an online reservation system that limited the numbers of visitors to enter during scheduled times and that controlled the number of visitors who could be allowed into each exhibit hall at any one time. These reservation systems were made available on a first come, first served basis. By mid-May, however, a total of 58 out of 137 all science museums (42%) still remained fully closed (the Korean Science Center and Museum Association, 2020). Figure 1 offers a timeline of events and describes the opening and closure of science museums since the pandemic was declared.

On June 29, 2020 the government implemented a new policy requiring people to practice social distancing. This policy gave authority to manage public facilities, such as museums, to local districts. In regions reporting large numbers of COVID-19 cases, many science museums have either remained fully closed or have closed again after initially re-opening. From May 29 to July 21, 2020, the GNSM was forced to close again and then was allowed to briefly re-open from July 22 to August 18, 2020. From August 19 to September 28, 2020 was again forced to close their doors to the public. Because the civil servant employees of GNSM were required to continue their administrative and project related work duties, the museum has been able to continuously develop and implement new educational materials made available free to the public on the museum website and their public YouTube channel.

The forced closures and partial/limited re-opening of science museums have had an enormous impact on how educators have begun to approach providing informal science education learning opportunities to the public. From September 29 until April 9, 2021, the GNSM was open and available to accept visitors in person using strict protocols to screen visitors and to collect information for contact tracing should it be necessary. However, during this time period, the number of visitors choosing to travel to the museum has much lower than normal as the public is also cautious about engaging in activities in enclosed, shared spaces. Museums classified as national museums, like the GNSM have an advantage compared to the many small, privately funded museums in Korea. Many private museums that closed at the start of the pandemic have still not re-opened. Several news articles have described similar fates for small and privately funded museums. Especially, private museums have been hard hit by the pandemic because they depend heavily on visitor entrance fees, revenue generated from museum rentals for special events, and fees for implementing education programming to maintain staff and facilities. In the Netherlands, for example, a survey from June 2020 revealed that one-quarter of museums expected to remain permanently closed (Network of European Museum Organizations, 2020). The pandemic is likely to have lasting effects on the informal science education field for decades to come.

At the time this paper is being prepared for final publication, Korea is experiencing a Fourth Wave of the pandemic. By mid-July 2021, Korea has been reporting the highest daily total of positive cases per day since the start of the pandemic (about 1,600 cases a day). It is widely expected that in the next few weeks, the museums may be forced to close again until further notice. In the sections that follow, we explore how the pandemic has impacted education at the largest, publicly funded science education museum in Korea. In the sections that follow, we introduce the museum and we describe how museum educators have responded to forced closures in the past. Specifically, we describe how museum educators have attempted to address issues of accessibility, for both the general public and visitors with special needs, through an expansion of online educational content and the development of outreach education programs targeting diverse groups of learners. After describing these responses, we raise some questions about the possibility for these responses to transform future teaching and learning practices in informal science education. For this study, we analyzed publicly accessible data from the museum website archives and from the museum YouTube channel. All resources are open for the public to access and analyze.

## Gwacheon National Science Museum response to the pandemic

The Gwacheon National Science Museum (GNSM) was selected to provide an example of how museum educators have responded to the pandemic here in Korea. The GNSM is located in the Seoul metropolitan area, where about half of Korea’s 50 million people live. According to Korean Ministry of Science and Information, Communication, Technology KMSICT (2020), the GNSM had over 2.5 million visitors in 2019, most of which (77.1%) were elementary and secondary students, and the remaining were adults (9.7%) and young children (13.2%) (GNSM, 2020a**)**. In addition to exhibition halls, the GNSM offers various science education courses, which visitors can register for as individuals or in small groups. Examples include an outdoor science camp and special exhibition programs, such as an exploration of astronomical observatories (GNSM, 2020b). GNSM also routinely provides professional training programs for educators and teachers (GNSM, 2020c). GNSM is one of the highest-funded national science museums in Korea, which has enabled the museum to be well developed and allows for the maintenance of several high-quality exhibitions, including the Future Imagination Hall, the Korean Science and Civilization Hall, and the Insectarium.

## The role of GNSM’s content sharing during the pandemic

Prior to the pandemic, GNSM had been using online media, such as YouTube and other online media (e.g., 국립과천과학관or gnsmscience). In addition, they made use of social media outlets, such as, Instagram (@scipia.gnsm), Facebook (scientorium), Twitter (@scientorium), and online blogs (GNSM, 2021; see for example blog.naver.com/nsm2010). The role of content in this online media was primarily for announcing and promoting educational programs and exhibitions. However, since the pandemic, YouTube has become a major education platform for GNSM as they have become very active to upload video content through this online medium. To better understand the ways in which GNSM’s use of this resource has changed, we analyzed and classified the content of YouTube videos posted before and during the pandemic. In comparing and describing the changes in the content, we could identify some ways in which the pandemic has impacted on GNSM’s posting practices. A discussion of these changes is intended to serve as a resource for considering how informal science education may continue to change and evolve in the future.

To begin, we classified all the videos (N = 658) on the GNSM YouTube channel uploaded from the time the channel started (March 18, 2013) through the end of the first year of the pandemic (December 31, 2020). Prior to the pandemic, the posting of content was irregular. For example, in February 2019, the GNSM uploaded only two videos, but in September 2019 they uploaded 39 videos. However, since the closure of the museum, posts have become more regular and now new content appears at least every 3 days.

To characterize the videos, we watched each video and determined how the videos could be sorted. We identified four broad categories: announcements, archival content, passive participation programs, and indirect experiential programs. Table 1 describes the different types of content posted before and after the pandemic and shows the change in number of posts in each category since the museum was first forced to close in February 2020.

**Table 1:** Classification of Gwacheon national science museum (GNSM) youtube channel videos

Types of videos	Description	Total number of videos (Percentage of content represented)		Overall change (±)
		03.2013 ~ 02.2020	02.25.2020 ~ 12.31.2020	
Announcements	Program promotions and advertisements	86 (26%)	41 (13%)	− 13%
Archival content	Recordings of events, awards, interviews, “behind the scenes” videos, and music videos	170 (51%)	32 (10%)	− 41%
Passive participation programs	Documentaries, science history, talk shows, lectures, and reading of science picture books	26 (8%)	129 (39%)	+ 31%
Indirect experiential programs	Exhibition tours, virtual reality (VR) tours, how-to videos, and ecological experiences, Live YouTube video broadcasting	48 (15%)	126 (38%)	+ 23%
	Total	330 (100%)	328 (100%)	

Since the museum was first forced to close, GNSM has uploaded as many videos in ten months (N = 328) to their YouTube channel as they did the entire seven years before the pandemic (N = 330). Table 1 shows the total number of videos uploaded and shows the percentage of content represented by the videos in each category.

Two categories significantly decreased in content and the other two greatly increased. The announcements category, which includes promotional and advertisement videos used to introduce and market exhibits and new events to the public decreased by more than half. An example of a video in this category includes an advertisement for a new exhibit called, *Let’s go find Galileo* (10.3–12.31) (https://www.youtube.com/watch?v=JG1gTdO3vFw). Archival video content, such as video of the *National Youth Science Song Contest* (https://www.youtube.com/c/gnsmscience/videos?view=0&sort=da&flow=grid), which primarily serves the purpose of documenting events held at the GNSM, also significantly decreased.

The next two categories saw significant increases. Videos characterized as passive participation programs (N = 129; + 31%) included those providing viewers with a one-way, non-interactive transmission of scientific knowledge that did not attempt to engage the viewer in activity, including thinking about questions, nor did these videos invite viewers to share ideas with the museum staff members or other viewers via the comments and responses section of the video. A documentary about Galileo Galilee (https://www.youtube.com/watch?v=GbXXRLWwJ74) is an example of videos in this category. The final category of video identified as content with indirect experiential programs also increased significantly compared to before (N = 126; + 23%). These videos provided viewers with scientific knowledge, but also invited viewers to interact by encouraging participation by taking part in specific activities or experiences. An exhibition tour video of the main hall in which the museum educator explained the science of springs and how they can be used to make waves encouraged viewers to reflect on what they were viewing and to anticipate what would happen. Another example included a video demonstrating a machine that is used to measure the velocity of sound (https://www.youtube.com/watch?v=ovdNE6BNTNc). Viewers were asked to consider how changes in sound corresponded to velocity of the waves being measured. These kinds of videos engaged viewers in thinking about the content they were seeing and prompted them to participate by trying to replicate activities at home.

Analysis of all of the video content revealed that prior to the pandemic, about one-quarter (26%) of all videos consisted of announcements use to promote new programs and to enhance marketing to engage more visitors to the museum. However, during the pandemic, these announcement videos represented only about 13% of all videos uploaded. Similarly, the archival video content of events, interviews, and exhibitions saw the greatest reduction with prior to the pandemic, these videos accounted for roughly half (51%) of all postings—but since the pandemic, these videos accounted for the smallest number of uploads. This suggests that the main purpose of the GNSM YouTube channel before the pandemic was to promote and archive videos of new events and activities (~ 77% of all content)

However, since the pandemic, passive participation video content represented more than one-third (39%) of all video content. An example includes the newly introduced *Science Talk Show*, during which museum educators invite a guest to talk about popular science topics, such as how science is portrayed in science fiction movies. For one *Science Talk Show*, a popular local YouTuber who makes science-related video content for his channel was invited to introduce science related scenes from films he enjoys. For this segment, the science educator and guest speaker discussed quantum mechanics in relation to the popular film the ‘Matrix’ (see https://www.youtube.com/watch?v=X3qvxCyo7js). These videos offer viewers’ insights about the scientific knowledge in the film, and also provides viewers with an opportunity to learn about how the museum science educator thinks about this film. This segment is popular and has been repeated several times with different guests who discuss the science in various films

Indirect experiential program content also increased significantly (from 15%) and represents more than one-third (38%) of all videos posted during the pandemic. An example of this kind of content is an *Exhibition Tour* video. In one *Exhibition Tour* video, a camera follows science museum educators as they show and explain different installations in the exhibits, which gives the viewer a “guided tour” of the science museum (https://www.youtube.com/watch?v=UoOkDG115Z4). This kind of content was rarely posted before the pandemic, but the number and variety of exhibition tour videos have rapidly increased during the pandemic. These *Exhibition Tour* videos serve as a substitute for visiting the museum in real life, which can allow people to indirectly experience the exhibits and to learn science.

Similar to the *Exhibition Tour* videos, before the pandemic, the GNSM often held on-site events to allow viewers to observe and discuss the science of natural phenomena. However, since the pandemic all on-site events have been canceled. In order to substitute these on-site events, GNSM has begun to hold such events via *YouTube live broadcasting*. Several popular examples have included celestial events, such as, a partial solar eclipse that took place in June 2021 (https://www.youtube.com/watch?v=J4TTmRYtEjw&t=107 s) which has had over 400,000 views, and the Gemini meteor shower which was broadcast in December 2020 (https://www.youtube.com/watch?v=xuQInq83-wA) with over 270,000 views. In each of these live YouTube video broadcasts, science educators described the phenomenon as it happened, they explained the related science concepts using additional resources (e.g., maps, graphs, or diagrams and demonstrations), and they responded to questions asked in real time in the chatroom.

Using these exhibition tours and live broadcasting of on-site events, the GNSM can continue to offer visitors a traditional museum education experience similar to what has been provided on site before the pandemic. However, a typical on-site event or even long-running exhibition may not have been able to accommodate the same number of visitors as was possible virtually. The increase in the number of passive participation and indirect experiential program content made available suggests GNSM sees a benefit to using the YouTube channel to provide virtual exhibition tours and special events for sharing scientific knowledge to visitors. If this kind of programming continues even after museums are able to fully re-open, it could provide improved access to science learning experiences for a wide variety of visitors who have faced barriers to physically visiting the museum in the past. This includes not only people who have been limited from accessing the museum due to physical limitations of museum spaces (lack of ramps, elevators, adequate seating and restroom facilities) needed for people with diverse physical needs, but also for people who cannot easily attend while managing young children or who cannot visit often because they live far away (e.g., people who live in rural areas, mountain regions, or remote islands in Korea). This indirect participation can also allow people who cannot afford the costs associated with traveling to and from the museum, or the entrance fees, or who cannot attend during museum business hours due to work or school conflicts.

If museums continue to be closed in the future, we can anticipate that the amount of content posted, and the types of content being developed may continue to expand. Expanded accessibility to informal science learning content through online media has resulted in an unexpected benefit of museum closures for the public. The expansion of museum content to online/virtual platforms may really serve to advance accessibility of informal science education learning opportunities in ways we could not anticipate before the pandemic. This is especially true for people with health problems or mobility issues who have long faced barriers to access museums. In the next section, we describe how GNSM museum educator’s growing awareness about the ways in which accessibility issues can limit informal science learning has contributed to some developments in their willingness and ability to provide science learning opportunities for people who have traditionally been excluded from public learning environments.

## Expanding learning opportunities for marginalized members of society

Accessing museum content online has the advantage of allowing visitors to enjoy learning about scientific content without time or space limitations and this is especially important for people who have difficulties to physically access science museums. A recent survey (Korean Ministry of Science and Information, Communication, Technology, 2019) has shown that Korean citizens have nearly universal access to high quality internet (99.7%) and that the vast majority of people (94.9 %) have internet-enabled device utilization in the home. However, even with widespread accessibility possible, if the video content created by museums is not developed with the universal design features necessary for people to be able to access and understand the content being posted—the impact will be limited. For example, research shows that videos without detailed subtitles, such as those explaining what kind of background music is playing, deaf/hard-of-hearing people may only be able to understand ~ 30–40% of video the content available on YouTube (Ha, 2020). Online content that fails to maximize features necessary for making content accessible for people who are deaf/hard-of-hearing or people are blind/low vision can further marginalize these communities from being able to access science educational opportunities being made available in new media formats.

To prevent the deepening of educational disparities in virtual learning environments, science museums will also need to pay attention to accessibility design issues. Unfortunately, even before the pandemic, few studies internationally or domestically, have focused on equity issues in science museums (Gi et al., 2019). Some researchers (Dawson, 2014) have been critical about the lack of attention paid to accessibility and integration of people with disabilities in informal science learning environments. In Korea, researchers (Im and Kim, 2009) have long argued the need for more research focused on how to improve the organization and design of educational programs and exhibits for people with disabilities and have encouraged the implementation of modifications necessary to make public facilities accessible to people with disabilities.

The GNSM was an early adopter of some of these suggestions and since 2009, has conducted the *Science Sharing Project* for people who have traditionally been marginalized in science learning environments. This project includes programs such as *Science Museum Visits You*, *Today Is a Science Museum Day*, *Science Hope Camp*, and *Remote Science Classroom*—all of which offer a variety of scientific and cultural activities for groups who lack opportunities to visit the GNSM regularly. Museum educators provide access to marginalized populations in Korean society by bringing museum resources and educational programming to rural areas and remote islands, to nursing homes and elderly day centers, and to schools and programs designed for students with special education needs or for multicultural students and families (such as North Korean defector students and their families or children with a Korean and a non-Korean parent). In 2019 alone, more than 5,000 people participated in the *Science Sharing Project* from 111 schools and organizations (GNSM, 2019).

However, due to the pandemic, these programs have been temporarily suspended as it is not safe for participants or museum educators to travel and interact with one another. To overcome these limitations, we learned from an informal interview with a GNSM insider that museum educators are currently working to repurpose the annual budget to develop alternative programming to continue to provide outreach to these communities. One example is the *Scibox Challenge* program, which provides science activity kits to students with special education needs so they can experience science activities in their homes. The GNSM is also developing programming to be able to upload science content videos to YouTube that include Korean Sign Language (KSL) for people who are deaf/hard-of-hearing to improve accessibility the museum science content. These offer two examples of initiatives that have resulted from a public health emergency that initially limited science museum access for all visitors. The pandemic provided GNSM educators opportunities to think more broadly about how to better develop resources that can support all members of the public to learn science. We anticipate the same may be true for other science educators around the world and we hope to see research publications in the future that describe the development and evaluation of implementation of these materials in hopes that informal science learning can become more inclusive in the future.

## Conclusions and implications for future research

In this paper, we described the current situation facing science museums in Korea, specifically the GNSM, and we have described the GNSM’s various responses to the pandemic for both the general public and for members of the public who have traditionally been marginalized from informal science learning environments. Similar to science museums globally, the GNSM has increased their online presence and they are working to develop strategies for educational outreach through the development of science kits, small-group on-site visits, and virtual online learning content. These changes have the potential to improve accessibility to informal science learning content for all visitors.

Until the GNSM can fully re-open, there will be significant changes for all visitors and educators who wish to participate in informal science learning experiences. Visitors will only be able to use the main entrance and they will need to check and record their temperature and provide their contact information in a visitor guest book that will be used for contact tracing if needed. No more than 10 people will be allowed to visit as a group and no more than 1,700 people can visit the museum in one day. During the weekend, visitors will need to use a website to make a timed-appointments and the exhibitions will only be partially open. Following these safety instructions is necessary for allowing public facilities to re-open, but this process will undoubtedly disrupt and change how visitors experience learning in science museums.

Researchers, science museums specialists, and the government need to consider how these changes will affect the educational activities of science museums and what kinds of policies or help is needed to overcome these constraints. Moving forward, we intend to conduct on-going research with science museum educators to learn how they can more effectively make use of online media and how to make learning opportunities accessible for all learners. We conclude this paper with a call to other researchers to consider how informal science learning may change in the future in response to continued global pandemics, as research currently shows there may more pandemics in the future. We offer the following questions for consideration:

- What kinds of policies at the government level can help to support informal learning centers to safely re-open?
- What roles do/should informal science learning institutes play during pandemics or crisis?
- Currently, most informal education theory points to the value for self-navigated learning and the importance of choice in what is experienced and for how long. However, if content is being curated only online, how will choice be affected, and what could be the benefits or limitations to the changes in how people choose content? How will the use of these virtual and online educational responses during the pandemic affect learners’ experiences in the long-term?
- If there are benefits to experiencing informal science institutes via online, will these resources and methods for providing virtual learning opportunities be maintained after the pandemic? What design aspects should be maintained or re-developed for virtual informal science learning in the future?
- What responses are needed from science education researchers and educators to be able to rapidly change learning environments to meet changing needs in the future?
- What kind of science teacher and museum educator training is needed to maximize student/visitor science learning during the pandemic?
- What are the benefits to shifting museum educational content to online learning platforms? Will this shift provide more access to informal learning opportunities for people who are disabled, live far from the museums, and have other barriers to in-person and on-site participation? What are the limits for virtual learning initiatives to support diverse learning populations?
- What kinds of exhibits could be created in this environment that are currently limited in brick-and-mortar museums?
- What opportunities exist for more global collaborations between museums in different countries? How might advances in virtual learning technologies allow museums in different countries to share exhibition access and to provide global educational experiences?
